# Pyrazinone Biosynthesis and Signaling—Myxo Style

**DOI:** 10.1021/acscentsci.4c00356

**Published:** 2024-03-12

**Authors:** Jeffrey D. Rudolf, Sandra Loesgen

**Affiliations:** † Department of Chemistry, University of Florida, P.O. Box 117200, Gainesville, Florida 32611, United States; ‡ Whitney Laboratory for Marine Bioscience, University of Florida, 9505 N. Ocean Shore Blvd., St. Augustine, Florida 32080, United States

**Figure 1:**
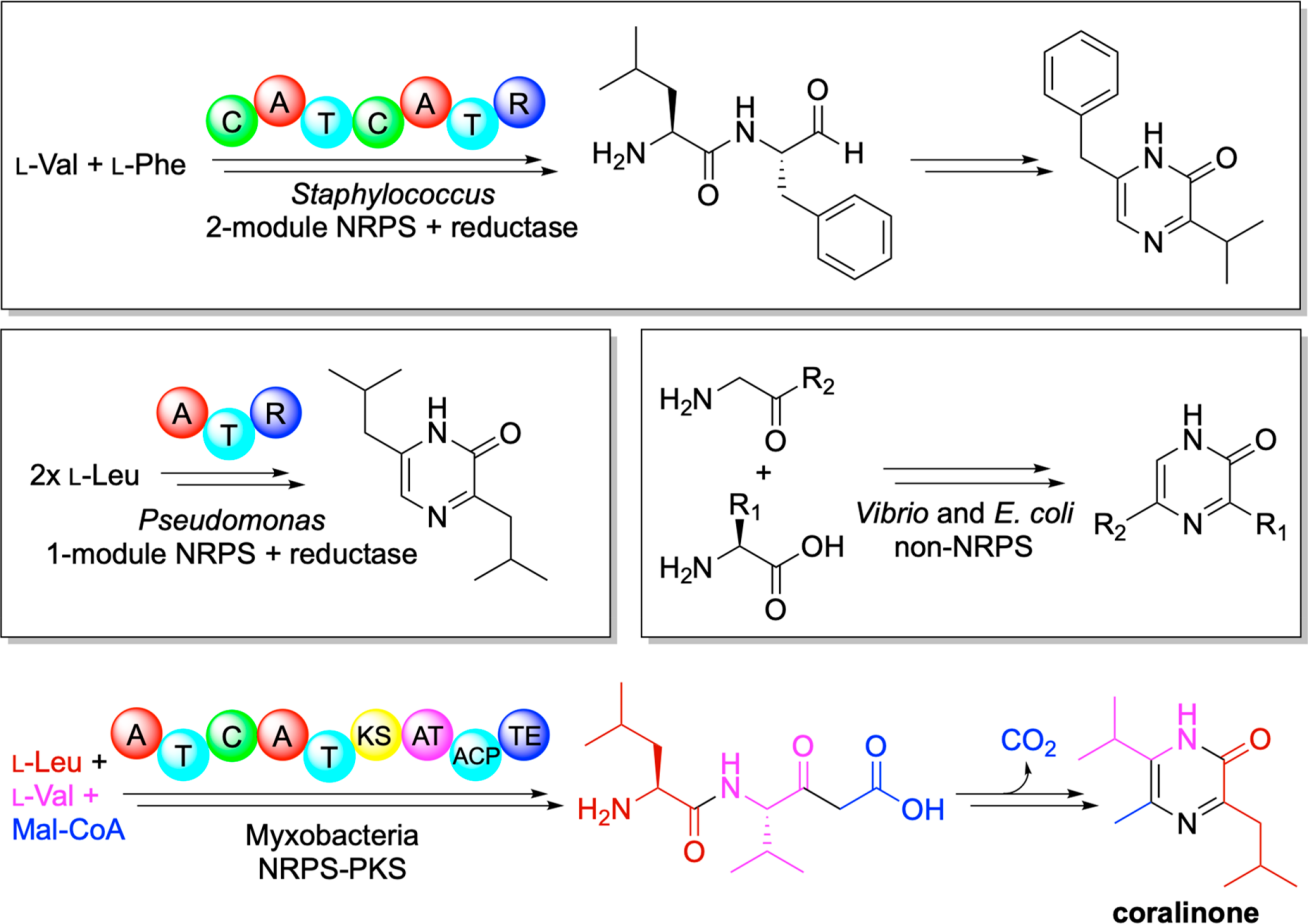
Distinct biosynthetic strategies of pyrazinone formation in bacteria. This study identifies a hybrid nonribosomal peptide synthetase/polyketide synthase in myxobacteria that forms trialkylated pyrazinones. Coralinone is a myxobacterial signaling molecule that promotes cellular aggregation.

Just as flight has evolved multiple times in different forms of life, so has signaling chemistry in microorganisms. In this issue of *ACS Central Science*, Wu, Zhang, Li, and co-workers describe the discovery and physiological function of coralinone, a 5-methylated pyrazinone signaling molecule, constructed by a single nonribosomal peptide synthase (NRPS)/polyketide synthase (PKS) gene in the myxobacterium *Corallococcus exiguus* SDU70 (see Figure 1).^1^

Pyrazinone scaffolds are a common motif in natural products with a broad range of biological properties, including kinase and protease inhibition and quorum sensing activity.^2^ They are found in all microbial domains of life, including the human microbiome, and play important roles as signaling molecules. Pyrazinones control biofilm formation in *Vibrio cholerae*, regulate the virulence of *Staphylococcus aureus*, and are involved in the pathogenesis of enterohemorrhagic Escherichia coli. The ecological roles of myxobacterial pyrazinones remain unknown, however.

While pioneering work by Bassler in the *Vibrio cholera* system established the importance of these quorum sensing molecules for biofilm formation and virulence,^3^ their impact extends beyond this and encompasses multiple domains of life. They found that small microbial pyrazine molecules also engage cellular receptors, RNA, proteins, and phages.^4,5^ Work by Crawford et al. identified the elusive autoinducer-3 as a pyrazinone (3,6-dimethylpyrazin-2(1*H*)-one) essential in the pathogenesis of enterohemorrhagic *E. coli* in the human microbiome.^6^ Moreover, the pyrazinone class of natural products exhibits various immunological effects on human tissue.^6^

This report adds myxobacteria, a chemically rich and large phylum, to the list of microbes engaging in pyrazine/pyrazinone biosynthesis and intra/interspecies signaling. The authors first found that coralinone (3-isobutyl-6-isopropyl-5-methylpyrazin-2(1*H*)-one) is uniquely constructed by single NRPS/PKS gene *corA* that installs the 5-methyl group. The pyrazinone core is often biosynthesized from the condensation of two amino acids through a multidomain nonribosomal peptide synthetase (NRPS) assembly line. But there’s a twist in myxobacteria.

Magarvey et al. and Fischbach et al. both characterized a two-module NRPS in *Staphylococcus aureus* that condenses two amino acids prior to reductive release of an aldehyde that can readily undergo nonenzymatic cyclization to yield the pyrazinone core (Figure 1).^6,7^ Li et al. more recently showed that a monomodular NRPS from *Pseudomonas* makes the same core structure.^7,8^ And in *Vibrio cholera* and *E. coli*, pyrazines/pyrazinones are formed from amino acids and aminoacetone, an oxidative byproduct of threonine (Figure 1).^3,6,9^

However, genome mining approaches using the antiSMASH tool^10^ with the genome of *Corallococcus exiguus* did not reveal a biosynthetic gene cluster like any of the other known pyrazinone-forming pathways. Instead, myxobacteria engage a single hybrid NRPS/PKS gene to cryptically add a malonyl or methylmalonyl unit, an evolutionarily elegant solution to provide a ketone via decarboyxlation rather than the usual aldehyde, to fashion trialkylated pyrazinones (Figure 1). The authors were able to produce coralinones *in vivo* using heterologous expression and *in vitro* biochemical reconstitution. This biosynthetic strategy appears to be conserved in myxobacteria, as 110 putative gene clusters encoding for trialkylated pyrazinones were found through genome mining. Interestingly, not all NRPS/PKS domain organizations were identical, implicating additional structural, and perhaps functional, diversity in the myxobacterial pyrazinone chemical space.

Coralinone promotes cellular aggregation of myxobacteria by enhancing the secretion of the extracellular matrix. Myxobacteria are known to secrete large amounts of the extracellular matrix, a hodgepodge of polysaccharides, proteins, and DNA, to support its social multicellular lifestyle. Coralinone was effective in inducing cellular aggregation not only of its native producer, *C. exiguus*, but also of the model organism, *Myxococcus xanthus*, indicating that its mode of action may be phylum-wide.

The cellular aggregation was not permanent, however. The authors noticed a peptidase encoded by *corB* genetically encoded near *corA*, the coralinone NRPS/PKS. Through both *in vitro* and *in vivo* assays, they found that CorB antagonized the agglutination effect of the pyrazinones by digesting membrane and extracellular proteins that act as the molecular glue of the extracellular matrix. In essence, myxobacteria self-regulate the dynamics of intra/interspecies cellular aggregation through the production of pyrazinones and the expression of corB, an interesting case of a self-regulatory growth system in bacteria.

Overall, the implications of coralinone biosynthesis and its ecological function could be expansive in the fields of microbial communication and myxobacterial chemistry and biology. These findings not only expand our understanding of pyrazinone biosynthesis and function but will open doors to unraveling the complex chemical ecology of these gliding bacteria and may provide enabling technologies to enhance chemical exploration or create genetically amenable strains.
